# Association of HDL subfractions with cardiovascular risk in Hungarian general and Roma populations

**DOI:** 10.1093/eurpub/ckac131.136

**Published:** 2022-10-25

**Authors:** P Pikó, NA Werissa, Z Kosa, J Sandor, I Seres, G Paragh, R Adany

**Affiliations:** 1 ELKH-DE-Public Health Research Group, University of Debrecen, Debrecen, Hungary; 2 Department of Health Methodology and Public Health, University of Debrecen, Debrecen, Hungary; 3 Department of Public Health and Epidemiology, University of Debrecen, Debrecen, Hungary; 4 Department of Internal Medicine, University of Debrecen, Debrecen, Hungary

## Abstract

**Background:**

High-density lipoprotein (HDL) cholesterol levels are inversely associated with cardiovascular risk (CVR). However, HDL cholesterol is not a homogeneous lipid and can be subdivided into subfractions, which are not uniformly associated with CVR. Among Roma populations, the prevalence of reduced HDL cholesterol levels and, consequently, that of cardiovascular diseases is very high. However, it is not known how this reduction affects the different HDL subfractions and whether changes in their representation are associated with changes in CVR.

**Methods:**

The study aimed to investigate whether there is a difference in the HDL subfraction profile between the Hungarian general (HG) and Roma populations and to determine the association of the different subfractions with the CVR estimated by the Framingham Risk Score (FRS) and the Systematic COronary Risk Evaluation (SCORE) algorithms. HDL cholesterol was separated using the Lipoprint system, which separates 10 subfractions into three classes: large HDL (HDL-L), medium HDL (HDL-I), and small HDL (HDL-S). Analyses were carried out on samples of 100 control subjects (50 Hungarian general and 50 Roma individuals with normal lipid profiles) and 277 individuals with reduced HDL-C levels.

**Results:**

Our results show that Roma has reduced levels of the overall HDL subfraction profile, with significant decreases in HDL-6, and -7. Regardless of the estimation method, elevated levels (in mmol/L) of HDL-1 to 3 and HDL-L were significantly associated with reduced risk. A higher representation (in %) of HDL-1 to 3 subfractions have a significant risk-reducing, while HDL-8 to 10 have a risk-increasing effect estimated by FRS.

**Conclusions:**

The results of our study show that levels of CVR protective HDL subfractions are significantly lower in Roma individuals and their reduced levels are associated with increased CVR, suggesting that the distribution of HDL subfractions contributes to the overall unfavourable CVR profile of Roma.

**Key messages:**

• Levels of HDL-6 and -7 subfractions were significantly lower in the Roma population than in the Hungarian general one.

• The HDL subfraction profile of the Roma population is associated with a higher cardiovascular risk among them.

